# Abdominal aortic calcification as an opportunistic marker of reduced CT-derived vertebral bone density in osteoporotic vertebral fractures

**DOI:** 10.1016/j.bas.2026.106133

**Published:** 2026-06-23

**Authors:** Biniam Melese Bekele, Sebastiano Vala, Ina Moritz, Yu-Mi Ryang

**Affiliations:** a Department of Neurosurgery & Center for Spine Therapy, Helios Klinikum Berlin-Buch, Germany; b Department of Neurosurgery, Klinikum rechts der Isar, Technical University Munich, Germany

**Keywords:** Osteoporotic vertebral fractures, Abdominal aortic calcification, Bone mineral density, Hounsfield units, Vascular calcification, Opportunistic imaging

## Abstract

**Background:**

Osteoporotic vertebral fractures (OVFs) frequently occur in patients without osteoporotic bone mineral density (BMD) on dual-energy X-ray absorptiometry (DXA), highlighting limitations of densitometry in capturing skeletal fragility. Abdominal aortic calcification (AAC) reflects cumulative cardiometabolic burden and may serve as a marker of impaired bone quality. This study evaluated the association between AAC and CT-derived vertebral bone density in patients with OVFs.

**Methods:**

In this retrospective cohort study, 172 patients with OVFs (median age 79 years, 72.7% female) underwent DXA, CT-based Hounsfield Unit (HU) assessment, and lateral lumbar radiography. AAC was quantified using the Kauppila 24-point score. Associations between AAC and bone density parameters were assessed using correlation and multivariable linear regression adjusted for age and sex. Sensitivity analyses included severe AAC (≥16) and low bone quality (HU ≤ 90).

**Results:**

AAC was inversely associated with HU in unadjusted analyses (r = −0.19, p = 0.032), with a stepwise decline across AAC categories (p = 0.038). This association was attenuated after adjustment (β = −0.68, 95% CI −1.55 to 0.20, p = 0.127), while age remained independently associated with lower HU (p = 0.030). No association was observed between AAC and DXA T-scores. Sensitivity analyses demonstrated a consistent but non-significant inverse relationship.

**Conclusion:**

AAC demonstrates a consistent inverse relationship with CT-derived vertebral bone density in OVF patients; however, this association is largely driven by age. These findings support AAC as an opportunistic imaging marker of skeletal fragility in older adults rather than an independent determinant of bone density.

## Highlights

• Abdominal aortic calcification is inversely related to CT-derived vertebral bone density in patients with osteoporotic vertebral fractures.
• This association is attenuated after adjustment for age, indicating that shared age-related processes largely drive the relationship.
• AAC may serve as an opportunistic imaging marker of impaired bone quality, particularly when DXA findings are inconclusive.

## 1 Introduction

Osteoporotic vertebral fractures (OVFs) represent a hallmark of advanced skeletal fragility and are associated with substantial morbidity, functional decline, and increased mortality (Son et al., 2023; Nazrun et al., 2014; Rizkallah et al., 2020). However, in clinical practice, OVFs frequently occur in individuals with non-osteoporotic areal bone mineral density (BMD) as assessed by dual-energy X-ray absorptiometry (DXA), particularly in older adults with degenerative spinal changes and vascular calcification (Mai et al., 2019; Jager et al., 2010). This discordance highlights the limitations of BMD as a sole measure of bone strength and suggests the contribution of systemic factors that are not captured by densitometry.

Osteoporosis and vascular calcification frequently coexist in aging populations and share overlapping pathophysiological mechanisms, including chronic inflammation, oxidative stress, and dysregulation of calcium–phosphate metabolism (Martini et al., 2023). Vascular calcification is an actively regulated process characterized by osteogenic differentiation of vascular smooth muscle cells and impaired inhibition of mineralization (Abedin et al., 2004; Massy et al., 2008). In parallel, osteoporosis is defined by uncoupled bone remodeling and progressive deterioration of trabecular microarchitecture, leading to reduced bone strength. These processes suggest a biological linkage between vascular and skeletal degeneration (Demer and Tintut, 2009).

Abdominal aortic calcification (AAC) represents a quantifiable imaging marker of systemic vascular calcification and cumulative cardiometabolic burden. It can be assessed opportunistically on lateral lumbar radiographs using validated semiquantitative scoring systems (Kauppila et al., 1997), allowing reproducible risk stratification without the need for advanced imaging. Despite growing interest in the bone–vascular interface, patients with manifest vertebral fractures remain underrepresented in studies evaluating AAC and skeletal health. Over the past decades, AAC has consistently been associated with adverse cardiovascular outcomes and mortality. Concurrently, observational studies have linked greater AAC burden to lower BMD and increased fracture risk, although effect sizes remain modest and heterogeneous across populations and fracture types (Kiel et al., 2001). A recent systematic review and meta-analysis pooling observational studies concluded that AAC is associated with lower BMD and higher fracture risk, supporting its role as a potential imaging biomarker at the intersection of cardiovascular disease and skeletal fragility (Gebre et al., 2023; Schousboe et al., 2026). However, data specifically addressing AAC severity in patients presenting with vertebral fractures, particularly using standardized radiographic scoring in combination with contemporary bone quality surrogates such as CT-derived trabecular attenuation (Hounsfield Units, HU), remain limited. The aim of the present study was to evaluate the association between AAC severity and bone status as assessed by DXA-derived T-scores and CT-derived HU in patients with osteoporotic vertebral fractures.

## 2 Methods

### 2.1 Study design and setting

This retrospective cohort study was conducted at a single high-volume spine center. The study was approved by the local ethics review board (Berlin Physicians Chamber, approval number Eth-SB-25-009 and conducted in accordance with the Declaration of Helsinki. Due to the retrospective design, the requirement for written informed consent was waived.

### 2.2 Patient population

Patients presenting to our institution between January 2021 and December 2024 with acute or subacute backpain and clinically suspected OVFs were screened for eligibility. Inclusion criteria were age ≥18 years, availability of BMD assessment using DXA, lumbar spine CT suitable for HU assessment, lateral lumbar spine radiography and clinical and radiographic evidence of acute OVFs indicating manifest osteoporosis. Manifest osteoporosis is defined in this study as the presence of at least one low-trauma (fragility) vertebral fracture. Patients with high-impact trauma, primary or metastatic spinal disease, spinal infection, spine instrumentation at the lumbar spine or inadequate imaging quality were excluded.

### 2.3 Assessment of abdominal aortic calcification

**Fig. 1:**
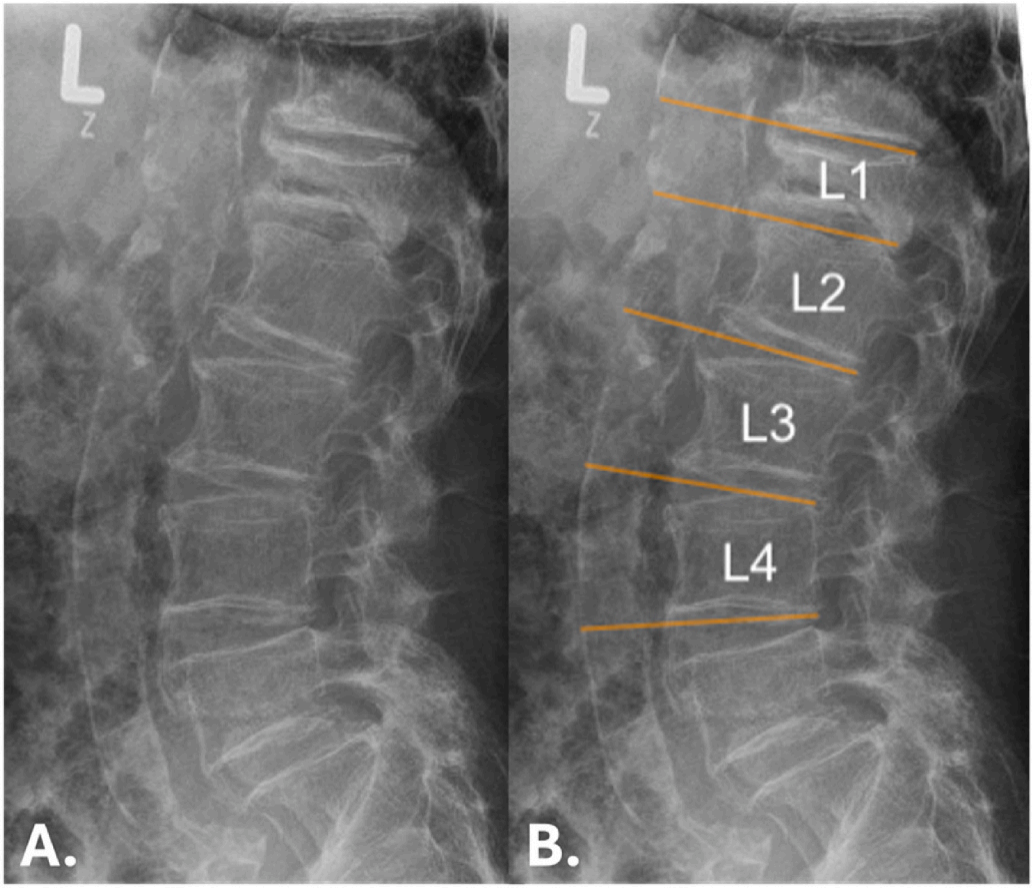
Assessment of abdominal aortic calcification (AAC) using the Kauppila scoring system. (A) Lateral lumbar radiograph. (B) Segmentation of the abdominal aorta adjacent to vertebral levels L1–L4. Calcification is evaluated separately along the anterior and posterior aortic walls at each vertebral level and graded from 0 to 3 based on the longitudinal extent of calcification. Segmental scores are summed to yield a total AAC score ranging from 0 to 24.

AAC was quantified on lateral lumbar spine radiographs using the validated Kauppila's 24-Point semiquantitative score (AAC-24) as previously described (Kiel et al., 2001). Briefly, the anterior and posterior wall of the abdominal aorta adjacent to L1-L4 were divided into four segments corresponding to the adjacent vertebral body. Calcified deposits within each segment were graded on a 0–3 scale based on their longitudinal extent relative to the vertebral body (0 = no calcification, 1 = calcification of <1/3 of the aortic wall length in that segment, 2 = calcification of 1/3 to 2/3 of the wall, and 3 = calcification of >2/3 of the wall) (see Fig. 1). Segmental scores were summed to yield a composite score ranging from 0 to 24. The 24-point scale has demonstrated high inter-rater reliability (intraclass correlation ∼0.8 in comparative studies) and slightly better consistency than simpler scores (Schousboe et al., 2006). The AAC severity was categorized as mild (total score: 0–4), moderate (total score: 5–15), or severe (total score: 16–24). Two readers blinded to clinical BMD values and fracture pattern independently assigned AAC scores.

### 2.4 BMD and HU assessment

BMD was assessed using standard dual-energy X-ray absorptiometry (DXA) protocols at the lumbar spine, femoral neck and hip (Lunar Prodigy; GE Healthcare, USA). T-scores were interpreted according to World Health Organization (WHO) criteria (Kanis, 1994). CT scan of the lumbar spine was performed using a 128-slice multidetector scanner (SOMATOM Definition AS+; Siemens Healthineers, Germany) with a slice thickness of 1.25 mm and tube voltage of 120 kV. Hounsfield unit (HU) measurements were obtained from at least two non-fractured vertebrae between L1 and L4. For each vertebra, three axial regions were analyzed: immediately below the superior endplate, at the mid-vertebral level, and just above the inferior endplate. Regions of interest (ROIs) were placed centrally within the trabecular compartment, avoiding cortical bone, endplates, venous plexus, and imaging artifacts, as previously described (Schreiber et al., 2011). Mean HU (mHU) values were calculated for each vertebra using the picture archiving and communication system (PACS) software (IMPAX EE R20; Agfa HealthCare, Belgium). The mHU across at least two non-fractured vertebrae was used for analysis.

### 2.5 Statistical analysis

Continuous variables are presented as mean ± standard deviation (SD) or median (interquartile range [IQR]) depending on distribution, and categorical variables as counts and percentages. Normality was assessed using the Shapiro–Wilk test and visual inspection of histograms. Group comparisons across AAC categories were performed using one-way analysis of variance (ANOVA) or Kruskal–Wallis tests, as appropriate, with post hoc pairwise comparisons adjusted using the Bonferroni method. Associations between AAC and BMD measures (CT-HU and DXA T-scores) were assessed using Spearman correlation coefficients. The primary analysis evaluated the relationship between AAC and HU using multivariable linear regression, with AAC entered as a continuous predictor and HU as the continuous outcome. Models were adjusted for age, and sex based on clinical relevance. Model assumptions were assessed by inspection of residual plots, evaluation of homoscedasticity, and testing for multicollinearity using variance inflation factors (VIF). Results are reported as regression coefficients (β) with 95% confidence intervals (CI). Sensitivity analyses included: (1) comparison of severe AAC (≥16) versus non-severe AAC using linear regression; and (2) logistic regression models evaluating the association between AAC and low bone quality, defined as HU ≤ 90. Adjusted odds ratios (OR) with 95% CI are reported. Interobserver reliability for AAC scoring was assessed using the intraclass correlation coefficient (ICC) based on a two-way random-effects model with absolute agreement. A two-sided p-value <0.05 was considered statistically significant. Analyses were performed using R (version 4.4.1; R Foundation for Statistical Computing, Vienna, Austria).

## 3 Results

### 3.1 Demographics

A total of 172 patients were included in this study with a median age of 79 (71 – 84) years and 125 (72.7%) were females. A total of 243 OVFs were seen. The majority of fractures 140 (60.1%) were located in the thoracolumbar junction. A total of 143 (58.9%) fractures were located in in the lumbar and 100 (41.1%) in the thoracic spine. A summary of the demographics is presented in Table 1 below.

**Table 1:** Demographics and comorbidities of patients.

Parameters	N = 172
Age (years)	79 (71 – 84)
Female, *n (%)*	125 (72.7%)
BMI (kg/m^2^)	23.5 ± 4.7
Comorbidities	
Hypertension, *n (%)*	115 (66.9)
Diabetes mellitus, *n (%)*	40 (23.3)
Dyslipidemia, *n (%)*	42 (24.4)
Statin use, *n (%)*	66 (38.4)
Chronic kidney disease, *n (%)*	82 (47.7)
Coronary artery disease, *n (%)*	19 (11)
Valvular heart disease, *n (%)*	9 (5.2)
Atrial fibrillation, *n (%)*	27 (15.7)
Chronic heart failure, *n (%)*	19 (11)
Laboratory parameters^a^	
Albumin _(35-52 g/l)_	35.1 ± 6.6
Hemoglobin _(13.5-17.2 g/dl)_	11.9 ± 2
Corrected Calcium _(2.2-2.65 mmol/L)_	2.4 ± 0.1
Phosphate _(0.87-1.45 mmol/L)_	0.9 ± 0.2
Creatinine _(<81_ _μmol/l)_	80.7 ± 28.5
BUN _(2.9-8.2 mmol/l)_	5.4 ± 2.7
Location of fracture, *n (%)*	
Thoracic spine	100 (41.1)
L1	40 (16.5)
L2	36 (14.8)
L3	30 (12.3)
L4	23 (9.5)
L5	14 (5.8)
Osteoporosis Parameters	
DXA-Scan	
T-score Lumbar spine	−1.6 ± 1.8
T-score Femoral neck	−2.0 ± 0.9
T-score Hips	−1.8 ± 1
mHU in CT	76.8 ± 32.4 (15.3 - 222.1ax)
AAC-24, *n (%)*	
Mild (0-4)	78 (45.4)
Moderate (5-15)	79 (45.9)
Severe (16-24)	15 (8.7)

AAC – Abdominal aorta calcification, BMI – Body Mass Index, BUN – Blood Urea Nitrogen, DXA – Dual-energy X-ray absorptiometry, mHU – Mean Hounsfield units.

a Laboratory normal values are depicted in brackets.

### 3.2 AAC-24, interobserver reliability and clinical parameters

A total of 78 (45.4%) patients had mild AAC-24 scores, 79 (45.9%) patients moderate and 15 (8.7%) severe AAC-24 scores. Interobserver agreement for AAC-24 scoring was excellent (ICC = 0.87, 95% CI 0.82–0.91, p < 0.001), indicating high reliability of radiographic assessment.

No significant differences were observed across AAC categories in serum hemoglobin, albumin, blood urea nitrogen, phosphate levels, or number of fractures. However, serum corrected calcium (p = 0.011) and creatinine levels (p = 0.003) increased significantly with greater AAC severity (see Table 2).

**Table 2:** Distribution of clinical, laboratory and bone density parameters across abdominal aortic calcification categories.

AAC-24	Mild^a^	Moderate^a^	Severe^a^	*p*
Laboratory parameters				
Albumin (g/l)	34.4 ± 6.04	34.9 ± 7.65	37.5 ± 3.48	0.328
Hemoglobin (g/dl)	11.8 ± 2.08	11.9 ± 2.06	12 ± 1.89	0.961
Corrected Calcium (mmol/L)	2.3 ± 0.09	2.36 ± 0.11	2.45 ± 0.06	*0.011*
Phosphate (mmol/L)	1.02 ± 0.19	0.98 ± 0.2	0.96 ± 0.08	0.775
Creatinine (μmol/l)	72.9 ± 23.7	88.1 ± 32.2	82.5 ± 19.7	*0.003*
BUN (mmol/l)	5.24 ± 3.35	5.52 ± 2.26	5.53 ± 2.29	0.878
Number of fractures, *n (%)*				0.216
Single	59 (34.3)	49 (28.5)	9 (5.2)	
Multiple^b^	20 (11.6)	29 (16.9)	6 (3.5)	
Bone density parameters				
mHU	82.7 ± 34.4	73.6 ± 30.8	61.9 ± 23.2	*0.0382*
T-score Lumbar spine	−1.52 ± 2.04	−1.62 ± 1.61	−1.64 ± 0.94	0.939
T-score Femoral neck	−1.98 ± 0.82	−1.94 ± 0.89	−2.34 ± 0.84	0.271
T-score Hips	−1.81 ± 0.91	−1.73 ± 1.11	−2.16 ± 0.89	0.342

BUN – Blood Urea Nitrogen, mHU – Mean Hounsfield units.

a Mild (total score: 0–4), moderate (total score: 5–15), or severe (total score: 16–24).

b Multiple defined as two and more OVFs.

### 3.3 Association between AAC and BMD

**Fig. 2:**
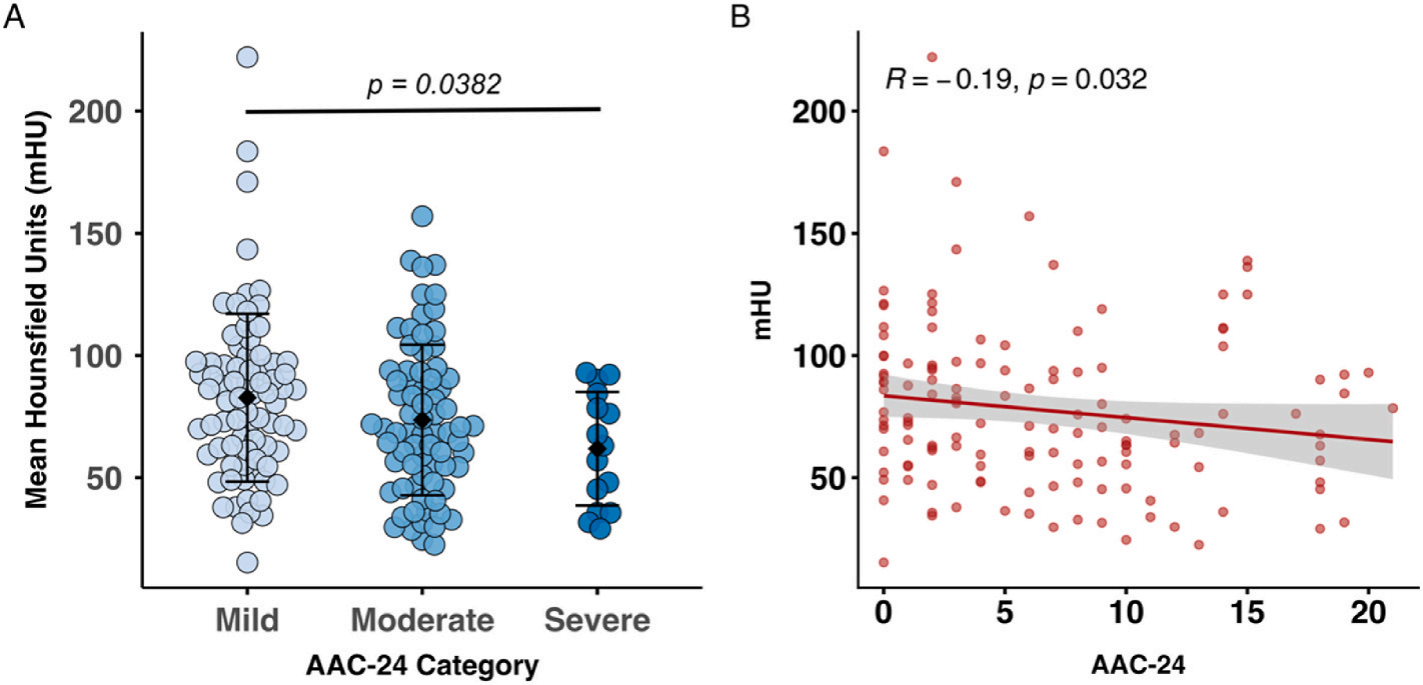
Association between aortic calcification and vertebral bone density. (A) Plot of mean Hounsfield Units (mHU) stratified by AAC-24 categories, showing a stepwise decrease in mHU with increasing calcification burden. (B) Correlation plot between AAC-24 and mHU showing a weak yet statistically significant inverse association.

Overall, 114 patients (62.3%) exhibited osteoporotic bone quality (mHU <90). Mean HU values differed significantly across AAC-24 categories, with progressively lower mHU observed in patients with higher AAC-24 scores (*p* = 0.038). AAC-24 demonstrated a statistically significant inverse correlation with mHU (r = −0.19, p = 0.032) (see Fig. 2).

DXA-defined osteoporosis (T-score ≤ −2.5) was present in only 49 patients (28.5%) at the lumbar spine, 43 patients (25.0%) at the total hip, and 44 patients (33.1%) at the femoral neck. No significant differences in DXA-derived T-scores (lumbar spine, total hip, or femoral neck) were observed across AAC categories (see Table 2). Similarly, no significant correlations were identified between AAC-24 scores and DXA T-scores (lumbar spine: *p* = 0.846, hip: *p* = 0.708, femoral neck: *p* = 0.308).

In age- and sex-adjusted linear regression analysis, AAC showed an inverse but non-significant association with CT-derived bone density (β = −0.68, 95% CI −1.55 to 0.20, *p* = 0.127). Age was independently associated with lower HU values (β = −0.60, 95% CI −1.14 to −0.06, *p* = 0.030), while sex was not significantly associated with HU (β = 2.84, 95% CI −8.22 to 13.9, *p* = 0.613).

Sensitivity analyses were conducted to further explore the relationship between AAC and bone quality. In sensitivity analysis comparing severe AAC (≥16) with non-severe AAC, severe calcification was associated with a 14.47 HU lower mean vertebral attenuation after adjustment for age and sex, although this did not reach statistical significance (β = −14.47, 95% CI −31.52 to 2.58, *p* = 0.096). In logistic regression analysis using osteoporotic bone quality (HU ≤ 90) as the outcome, AAC was not independently associated with risk after adjustment for age and sex (OR = 1.03 per point increase, 95% CI 0.97–1.09, *p* = 0.375). Across all models, AAC consistently demonstrated an inverse but non-significant association with HU. These findings support a stable directional relationship between vascular calcification and bone density. However, the attenuation of the association after adjustment indicates that much of the observed relationship is driven by age, suggesting that AAC may be more reflective of shared biological aging and frailty rather than an independent determinant of bone density.

## 4 Discussion

This study demonstrates that AAC is associated with reduced vertebral bone density assessed by CT-derived HU in patients with osteoporotic vertebral fractures, while no relationship was observed between AAC and DXA-derived T-scores. These findings highlight a clinically relevant discordance between conventional densitometry and structural bone density in a population characterized by advanced skeletal fragility. A consistent stepwise decline in HU was seen across increasing AAC severity. However, this effect was no longer statistically significant after adjustment for age, indicating that much of the observed relationship is driven by shared age-related processes.

The inverse association between AAC severity and HU supports the concept of a shared pathophysiological axis linking vascular calcification and skeletal deterioration. Multiple observational studies and meta-analyses suggest that AAC is associated with lower BMD and higher fracture risk with effect sizes generally modest but reproducible across populations. In both cross-sectional and prospective cohorts, individuals with more extensive abdominal aortic calcifications tend to have lower BMD and higher fracture rates (Gebre et al., 2023, 2026; Szulc et al., 2015). A large meta-analysis of 27 studies has shown that the presence of significant AAC corresponded to roughly a 1.7-fold higher risk of vertebral and non-vertebral osteoporotic fractures fragility (Gebre et al., 2023). A prospective study of osteoporotic fractures has described the association between severe AAC and an OR of 2.3 risk of new vertebral fractures and an almost threefold increase in hip fracture risk (Szulc et al., 2015). Similarly, a 5-year Chinese cohort study of postmenopausal women reported that the highest aortic calcification scores were associated with a greater incidence of both vertebral and non-vertebral fragility fractures even when adjusted for age and other risk factors (Zhou et al., 2014). These findings have been consistently observed across Western and Asian populations, underscoring the robust relationship between advanced aortic calcification and skeletal fragility. Our findings extend this literature by focusing specifically on patients with manifest osteoporosis and incorporating CT-derived HU as measurement for BMD. In contrast to DXA, which provides a two-dimensional estimate of areal BMD, HU measurements capture aspects of trabecular microarchitecture and mineralization that are more directly related to mechanical strength. The observed stepwise decline in HU across AAC categories is therefore biologically plausible and consistent with emerging evidence that CT-based metrics outperform DXA in fracture risk discrimination.

The biological and clinical underpinnings of this association are still an area of active investigation. AAC and osteoporosis likely share common etiologies, including estrogen deficiency, chronic inflammation, oxidative stress, and dysregulated mineral metabolism (Demer and Tintut, 2009; Cannata-Andia et al., 2011). In line with this, we observed increasing levels of serum corrected calcium and creatinine across AAC categories, suggesting that vascular calcification reflects systemic alterations in calcium–phosphate balance and renal function. Additional mechanisms may include impaired vascular supply to the vertebrae due to adjacent aortic calcification, potentially affecting bone remodeling (Cannata-Andia et al., 2011; Hjortnaes et al., 2010). Epidemiologically, higher AAC scores have been associated with lower BMD, increased fracture risk, and broader functional decline, including reduced muscle strength and increased fall risk (Demer and Tintut, 2009; Gebre et al., 2023). Together, these findings support the concept of AAC as an integrated marker of accelerated aging, identifying patients at elevated risk for skeletal fragility.

The attenuation of the AAC–HU association after adjustment is a key finding and underscores the central role of age in this relationship. While vascular calcification and skeletal deterioration share common biological pathways, both processes are fundamentally driven by aging. Importantly, the present findings do not refute the existence of a bone–vascular axis but rather suggest that AAC operates within a broader framework of age-related systemic decline. This interpretation is supported by prior studies demonstrating heterogeneity in the strength and independence of the association (Wang et al., 2010a; Samelson et al., 2007; Flipon et al., 2012)–(Wang et al., 2010a; Samelson et al., 2007; Flipon et al., 2012). Earlier work by Wang et al. (2010), for instance, reported no significant association between baseline AAC and subsequent fractures after adjusting for age (Wang et al., 2010b). Collectively, these findings indicate that age should not be viewed solely as a confounder but as a central determinant of both vascular and skeletal pathology. In this context, AAC should be interpreted less as an independent causal factor and more as an integrated marker of biological aging and cumulative disease burden. The attenuation observed in adjusted models therefore reflects the integration of AAC within broader biological aging processes rather than negating the clinical relevance of the observed association.

From a clinical standpoint, the key implication of this study is not whether AAC independently predicts bone density, but rather these findings suggest that AAC may serve as an opportunistic imaging biomarker of impaired bone quality in older patients despite inconclusive or misleading DXA findings. AAC can be assessed on standard lateral lumbar radiographs without additional cost, additional radiation exposure, or workflow disruption and can be used as a readily available imaging signal to identify patients at risk of poor bone quality**.** The consistent inverse relationship observed across analyses suggests that increasing AAC burden should prompt clinicians to consider further evaluation of bone quality, even in patients without overtly osteoporotic DXA values.

This study benefits from a well-characterized cohort of patients with manifest osteoporosis with clinically relevant vertebral fractures, standardized assessment of AAC using a validated semiquantitative scoring system, and the inclusion of CT-based bone quality metrics. The excellent interobserver reliability supports the robustness of the AAC measurements. While CT-based quantitative methods such as Agatston or volumetric scoring provide more precise assessment of vascular calcification, these require dedicated software and standardized acquisition protocols (Devia et al., 2023). In contrast, the Kauppila score enables opportunistic and reproducible assessment on routine lateral spine imaging, making it particularly suitable for clinical screening applications. However, several limitations warrant consideration. The retrospective design and single-center setting may introduce selection bias and limit generalizability. The cross-sectional nature of the analysis precludes causal inference. Furthermore, the relatively small number of patients in the severe AAC category may reduce statistical power for subgroup comparisons.

## 5 Conclusion

In patients with OVFs, AAC is associated with reduced vertebral bone density as measured by CT-derived HU. While the relationship between AAC and bone density is influenced by age, the consistent inverse trend observed across analyses supports the use of AAC as a radiographic marker to prompt further evaluation of bone quality. Incorporation of AAC assessment from routine imaging may enhance identification of patients with impaired bone quality, particularly when combined with CT-derived metrics. Future longitudinal studies are required to clarify the prognostic and causal relevance of the bone–vascular relationship.

## Declaration of generative AI and AI-assisted technologies

During the preparation of this work the authors used ChatGPT to improve sentence structure and proofing. After using this tool/service, the authors reviewed and edited the content as needed and take(s) full responsibility for the content of the published article.

## Declaration of competing interest

The authors declare the following financial interests/personal relationships which may be considered as potential competing interests:Yu-Mi Ryang has served as a speaker and lecturer at various symposiums organized by Brainlab®, DePuy Synthes (Johnson & Johnson), icotec AG, Medtronic. She is also involved in annual courses and trainings and committee member or chair in national and international societies such as AO Spine, the German Spine Society (DWG), the European Association of Neurosurgical Societies (EANS), and EUROSPINE. If there are other authors, they declare that they have no known competing financial interests or personal relationships that could have appeared to influence the work reported in this paper.

## Abbreviations:

AAC – Abdominal Aortic Calcification, AAC-24 – 24-point Kauppila Abdominal Aortic Calcification Score, BMD – Bone Mineral Density, BMI – Body Mass Index, BUN – Blood Urea Nitrogen, CI – Confidence Interval, CT – Computed Tomography, DXA – Dual-energy X-ray Absorptiometry, HU – Hounsfield Units, ICC – Intraclass Correlation Coefficient, IQR – Interquartile Range, mHU – Mean Hounsfield Units, OVF – Osteoporotic Vertebral Fracture, ROI – Region of Interest, SD – Standard Deviation.
